# An Act to Regulate the Practice of Dentistry in Arizona

**Published:** 1894-01

**Authors:** 


### DENTAL LEGISLATION.

### An Act to Regulate the Practice of Dentistry in Arizona.

*Be it Enacted by the Legislative Assembly of the Territory of Arizona:*

SECTION 1. That it shall be unlawful for any person, who is not at the time of the passage of this Act, engaged in the practice of dentistry in this Territory, to commence such practice unless such person shall have received a license from the Board of Examiners, as hereinafter provided for.

SEC. 2. The Governor of the Territory shall appoint, after the passage of this Act, five (5) skilled dentists of good repute, residing and doing business in the Territory, who shall constitute a Board of Registration in Dentistry.

But no person shall be eligible to serve on said Board unless they have been regularly graduated from some reputable dental college, duly anthorized to grant degrees in dentistry, or who shall have been actively engaged in the practice of dentistry for a period of ten (10) years previous to appointment.

SEC. 3. The length of term for which the members of said Board shall hold office shall be three (3) years, except that two of the members of the Board first to be appointed under this Act shall hold office for the term of one (1) year, two for the term of two (2) years, and one for the term of three (3) years respectively, and until their successors shall be duly appointed and qualified.

In case of a vacancy occurring in said Board, such vacancy shall be filled by the Governor in conformity with Section 2.

SEC. 4. Said Board shall choose one of its members President and one Secretary and Treasurer, and it shall meet at least once a year, and oftener if it shall be deemed necessary.

Four of said Board shall constitute a quorum.

The proceedings of said Board shall at all reasonable times be open to public inspection.

SEC. 5. It shall be the duty of each person now engaged in the practice of dentistry in this Territory, to within ninety (90) days after the passage of this Act, to send an affidavit to the Secretary of said Board, setting forth his or her name, place of business, post office address, the length of time they have been engaged in the practice of dentistry in this Territory; if a graduate of a dental college, state the name of college; and shall pay to the Treasurer of said Board the sum of Five Dollars ($5), for which they shall receive from said Board a practitioner's certificate.

On failure to comply with the provisions of this section they shall be required to appear before the Board and be examined by said Board.

SEC. 6. It shall be the duty of all persons not holding diplomas, who wish to engage in the practice of dentistry in this Territory, after the passage of this Act, to appear before said Board at a regular meeting and pay into the Treasury of said Board the fee of Twenty-Five Dollars ($25), not returnable, and stand an examination by said Board in operative and prosthetic dentistry, and all the branches taught in a reputable dental college, and if such applicants pass an examination satisfactory to said Board, said Board shall issue to said applicant a license which will entitle him or her to practice dentistry in this Territory.

SEC. 7. It shall be the duty of all persons holding diplomas, who wish to engage in the practice of dentistry, after the passage of this Act, to present or send to the Secretary at the regular meeting of said Board an affidavit and diploma with fee ($5), not returnable, and after said Board being satisfied that said diploma belongs to said applicant, and that it was issued in good faith by a reputable dental college, said Board shall issue to said applicant a certificate of registration for said diploma.

SEC. 8. All persons receiving a certificate to practice under this Act shall register his or her certificate with the County Recorder of the county in which he or she resides, and shall pay to the County Recorder for such registration, the sum of Two Dollars ($2).

Any failure on the part of any person holding such certificate to comply with the first part of this section within thirty days (30) after receiving certificate, shall forfeit said certificate, and any certificate once forfeited shall not be returned by said Board until applicant shall have paid to said Board the fine of Twenty-Five Dollars ($25).

It shall be the duty of each County Recorder, to forward to the Secretary of said Board the names of all persons having registered their certificates with them.

SEC. 9. It shall be the duty of said Board to cause to be kept a record of all its proceedings, and the names and addresses of all persons qualifying under this Act.

An annual report of the same shall be rendered to the Governor.

All moneys received by the Secretary under this Act shall be used for the legitimate expenses of said Board, but in no case shall any money of the Territory be used for that purpose.

SEC. 10. Any person or persons violating any provisons of this Act shall be deemed guilty of a misdemeanor, and upon conviction shall be fined not less than One Hundred Dollars ($100) nor more than Two Hundred Dollars ($200), or confined six months in the county jail, or both, for each and every offense.

All fines recovered under this Act shall be paid into the common school fund of the county in which such conviction takes place.

SEC. 11. It shall be the duty of the Prosecuting Attorney of each county to prosecute such cases when brought to his knowledge.

SEC. 12. That nothing in this Act shall be construed so as to interfere with the rights and privileges of resident physicians and surgeons in the discharge of their professional duties.

SEC. 13. This Act shall take effect immediately after its passage.

Approved April 3, 1893.

